# Changes in and significance of platelet function and parameters in Kawasaki disease

**DOI:** 10.1038/s41598-019-54113-1

**Published:** 2019-11-27

**Authors:** Xiaolan Zheng, Wenchao Wu, Yi Zhang, Gang Wu

**Affiliations:** 1 Department of Pediatrics, West China Second Hospital, Sichuan University, Chengdu, Sichuan 610041 China; 2 Key Laboratory of Birth Defects and Related Diseases of Women and Children (Sichuan University), Ministry of Education, West China Second Hospital, Sichuan University, Chengdu, Sichuan 610041 China; 3 West China Medical School, Sichuan University, Chengdu, Sichuan 610041 China; 4 Laboratory of Cardiovascular Diseases, Regenerative Medicine Research Center, West China Hospital, Sichuan University, Chengdu, China

**Keywords:** Diagnostic markers, Vasculitis, Platelets

## Abstract

Kawasaki disease (KD) is a systemic febrile, inflammatory vascular disease of unknown etiology. The coronary artery abnormality (CAA) caused by KD has become the most commonly acquired heart disease in children. Initial treatment of intravenous immunoglobulin (IVIG) can reduce the incidence of CAA. Thrombocytosis is common during the course of KD, but changes in and significances of platelet function and parameters are unclear. In this study, we enrolled 120 patients, including 40 patients with KD, 40 febrile controls, and 40 afebrile controls. The platelet function was assessed using the platelet function analyzer (PFA)-200. Platelet parameters, including platelet count (PLT), mean platelet volume (MPV), platelet distribution width (PDW), and platelet hematocrit (PCT) were measured. In the febrile period, the PDW and MPV were lower in KD patients (P < 0.05). The platelet function did not change significantly during the febrile period of KD but weakened in the defervescence phase. No significant differences between the CAA and normal groups, and between IVIG resistance and response groups. The diagnostic cutoff value of the PDW level for predicting KD was 10.85 fL with a sensitivity of 55% and a specificity of 77.5% (area under curve (AUC) = 0.690, 95% confidence interval (CI): 0.574–0.806, P < 0.01). Besides, the MPV level was 9.55 fL with sensitivity of 75% and specificity of 70% (AUC = 0.733, 95%CI: 0.620–0.846, P < 0.001). This is the first longitudinal study of platelet function changes in KD patients using PFA-200. Besides, lower PDW and MPV may be available markers for early diagnosis of KD.

## Introduction

Kawasaki disease (KD) is a systemic small and medium-sized vascular inflammatory disease of unknown etiology, which mainly affects children under five years of age^1^. The coronary artery abnormality (CAA) caused by KD has become the most commonly acquired heart disease in children^2^. Timely initiation of treatment with intravenous immunoglobulin (IVIG) can reduce the incidence of CAA from 25% to ≈4%^3^. At present, the view about the etiology of KD is that genetically susceptible children are exposed to an unknown factor that triggers an immune response aimed at parts of the artery^1–3^. So far, KD is diagnosed based primarily on the clinical features; there are no specific laboratory tests for the early identification and diagnosis^4^. However, delay accurate diagnosis may cause increased mortality and morbidity from complications of KD^5^. Previous studies indicated that thrombocytosis is common in the subacute stage of KD patients^6–8^. Meanwhile, platelet activation was closely related to the incidence of CAA^6^. Therefore, platelet function testing is a crucial element to comprehend the biology, pathology, and diagnosis of KD. However, testing of platelet function, whether using light transmittance aggregometry (LTA) or whole blood aggregometry (WBA), is complex, specialized, and time-consuming^9^. The platelet function analyzer (PFA)-100 (Siemens Healthcare, Marburg, Germany) is a system used globally and has recently been upgraded to the PFA-200^10^. However, it was notable that no study has examined the platelet function in KD patients using PFA-200.

PFA-200 testing involves an easily automated process that takes a short time to get the test results^11^, which is a high-shear system using whole blood rather than platelet-rich plasma^12^. It simulates the characteristics of physiological platelet function^13^ and measures the closure time (CT) required for platelets to plug an aperture simulating an injured vessel^14^, after platelet activation by relevant stimuli, namely collagen and epinephrine (EPI), or collagen and adenosine diphosphate (ADP)^15^. The maximum possible CT is 300 s, and values above 300 s corresponding to non-closure are considered invalid^16^. Aspirin influences the EPI value, whereas the ADP is unaffected. Thus, if the CT value of EPI in the test is prolonged, while that of ADP is normal, then it can be inferred that the CT value of EPI is prolonged due to aspirin. However, the prolonged CT values of EPI and ADP indicate that the prolonged CT value of EPI may be attributed to other causes of platelet dysfunction^17^.

The purpose of our study was to investigate the changes and significance of platelet function and parameters in KD patients. Specifically, we aimed to (1) evaluate the usefulness of platelet function and/or parameters as diagnostic biomarkers for KD, (2) evaluate the relationship between platelet function and/or parameters, and CAA in patients with KD, (3) evaluate the relationship between platelet function and/or parameters, and IVIG resistance in children with KD, and (4) evaluate the changes of platelet function and parameters during various stages of KD.

## Results

### Patient characteristics

Of the 40 KD patients enrolled in the present study, two were incomplete KD; three were resistant to the initial IVIG therapy. Besides, CAAs occurred in four KD patients during the febrile phase and continued into the convalescence stage. All CAAs were the only dilation without aneurysms. At last, three patients without previous CAA were lost to follow-up during the convalescence stage. Of the 40 patients in the fever control group, there were 18 cases of infectious mononucleosis, 13 of febrile pneumonia, and nine of acute tonsillitis. Of the 40 children in the afebrile control group, 21 patients had afebrile pneumonia, five had acute gastroenteritis, and 14 had acute bronchitis.

The differences in age and gender between the KD group and control groups were not statistically significant for comparability. The demographic, clinical, and laboratory data at the time of admission of patients in the three groups were shown in Table 1. In this phase, KD patients were not yet treated with IVIG and were in the febrile phase, as were patients in the febrile control group.

**Table 1:** Basic demographic, clinical, and laboratory data at the time of admission of the patients in the three groups.

Patient Characteristics	KD (n = 40)	Febrile control (n = 40)	Afebrile control (n = 40)	P
Age (years) (range)	3.01 ± 1.69 (0.50–7.90)	3.19 ± 2.04 (0.20–7.90)	3.19 ± 2.24 (0.20–8.4)	0.895
Male/female (%)	19 (47.50)/21 (52.50)	19 (47.5)/21 (52.50)	17 (42.5)/23 (57.50)	0.877
WBC (×10^9^ cells/L)	13.25 ± 3.11	11.95 ± 6.24	7.51 ± 2.42	<0.001
HB (g/L)	111.43 ± 8.78	114.38 ± 13.79	120.75 ± 10.64	0.001
PLT (×109 cells/L)	332.98 ± 103.71	326.88 ± 151.43	313.6 ± 118.58	0.782
PDW (fL)	10.16 ± 1.43	11.53 ± 2.34	11.1 ± 1.96	0.007
MPV (fL)	9.34 ± 0.75	10.15 ± 1.14	9.84 ± 0.99	0.001
PCT (%)	0.33 ± 0.13	0.32 ± 0.13	0.32 ± 0.12	0.944
CRP (mg/dL)	82.52 ± 41.97	24.93 ± 27.87	3.91 ± 3.53	<0.001
EPI (s)	100.4 ± 20.80	108.85 ± 28.13	87.83 ± 14.62	<0.001
ADP (s)	80.25 ± 16.17	83.5 ± 19.00	74.38 ± 10.33	0.033

KD = Kawasaki disease, WBC = white blood cel, HB = hemoglobin, PLT = platelet count, PDW = platelet distribution width, MPV = mean platelet volume, PCT = platelet hematocrit, CRP = C-reactive protein, EPI = closure time of collagen and epinephrine, ADP = closure time of collagen and adenosine diphosphate.

### Comparison of platelet function and parameters between the KD and control groups

**Figure 1:**
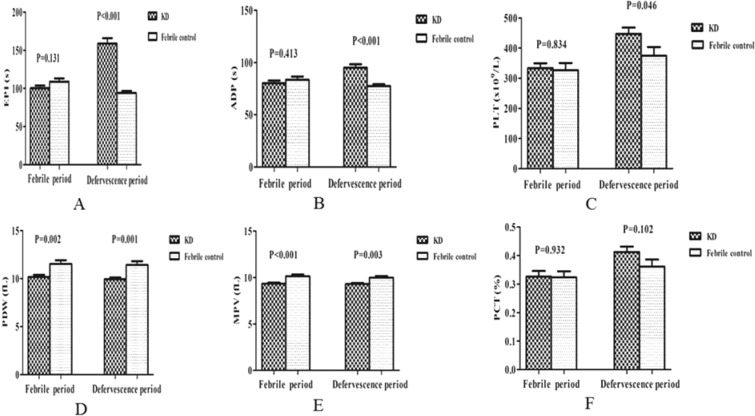
Comparison of platelet function and parameters between the KD group and the febrile control group. (**A**) Comparison of EPI between two groups. (**B**) Comparison of ADP between two groups. (**C**) Comparison of PLT between two groups. (**D**) Comparison of PDW between two groups. (**E**) Comparison of MPV between two groups. (**F**) Comparison of PCT between two groups.

**Figure 2:**
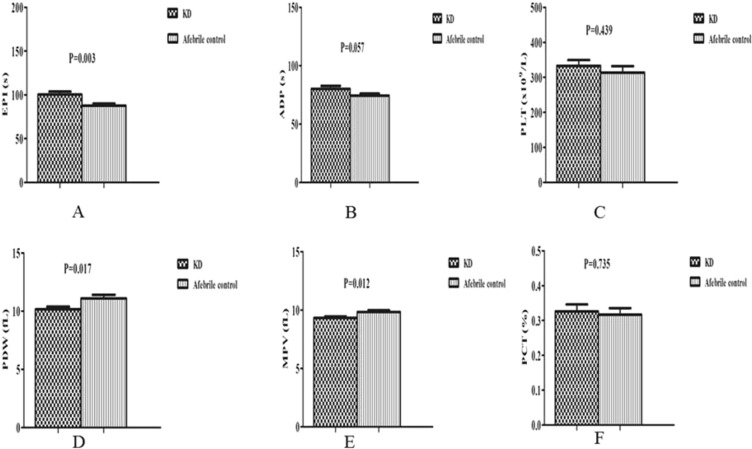
Comparison of platelet function and parameters between the KD group and the afebrile control group. (**A**) Comparison of EPI between two groups. (**B**) Comparison of ADP between two groups. (**C**) Comparison of PLT between two groups. (**D**) Comparison of PDW between two groups. (**E**) Comparison of MPV between two groups. (**F**) Comparison of PCT between two groups.

Platelet function (EPI, ADP) and some platelet parameters (PDW, MPV) showed significant differences among the three groups (Table 1). Therefore, we separately compared the data regarding platelet function and platelet parameters between the KD group and febrile control group (Fig. 1), and between the KD group and afebrile control group (Fig. 2).

Compared with the febrile group, both EPI and ADP were not significantly different in the febrile period (*P* > 0.05, Fig. 1A,B), but both were significantly higher in the defervescence period in the KD group (*P* < 0.001, Fig. 1A,B). PLT was not significantly different in the febrile period (*P* > 0.05, Fig. 1C), but was significantly higher in the defervescence period in the KD group (*P* < 0.05, Fig. 1C). PDW and MPV were significantly lower in the KD group during both the febrile and defervescence periods (*P* < 0.05, Fig. 1D,E). PCT was not significantly different during the two periods between the KD group and febrile group (*P* > 0.05, Fig. 1F).

Compared with the afebrile group, EPI was significantly higher in children with KD (*P* < 0.01, Fig. 2A). ADP showed no significant differences between the two groups (*P* > 0.05, Fig. 2B). There was no significant difference between the KD patients and afebrile group in terms of PLT and PCT (*P* > 0.05, Fig. 2C,F). PDW and MPV were significantly lower in the KD group (*P* < 0.05, Fig. 2D,E).

**Figure 3:**
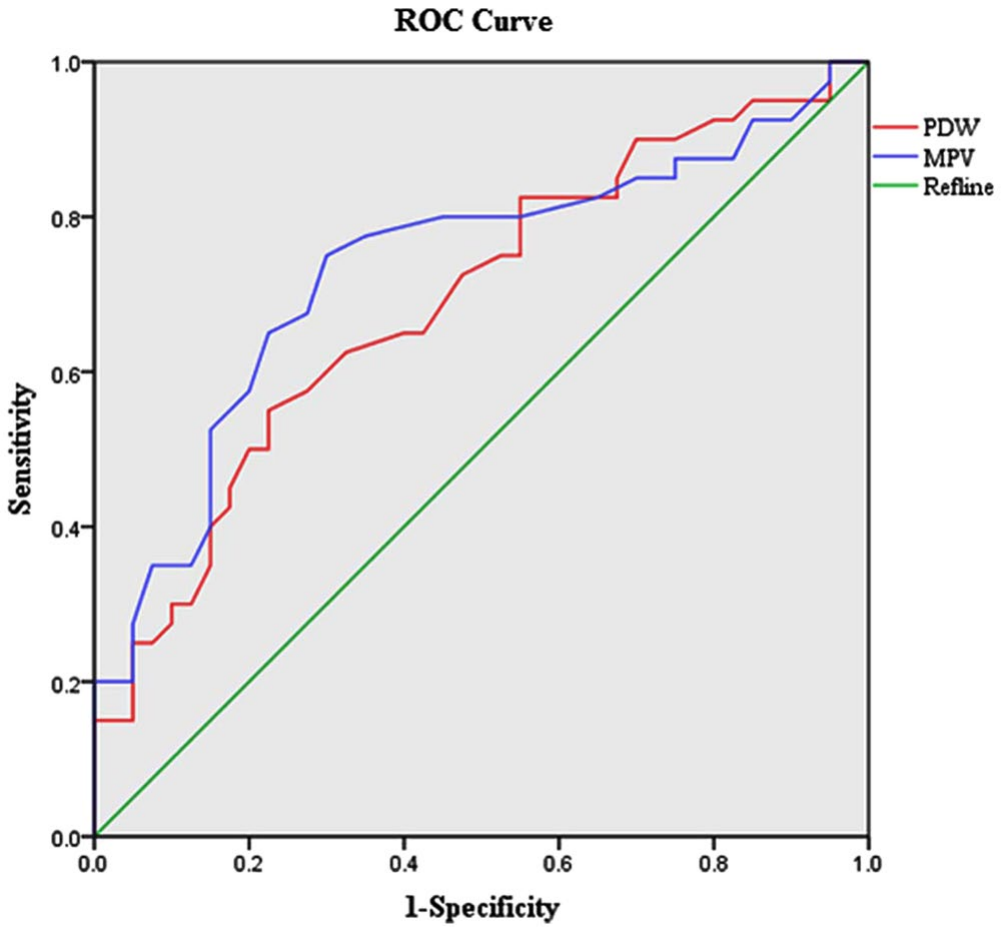
ROC curves of PDW and MPV in the prediction of KD.

After the above statistical comparison of platelet function and platelet parameters between the KD group and febrile control group, we selected two indicators (PDW, MPV), which had statistically significant differences between the KD group and febrile control group in the febrile period for the receiver operating characteristic (ROC) curve analysis. According to the ROC analysis, the diagnostic cutoff value of the PDW level on admission for predicting KD compared to that in patients with common febrile illness was 10.85 fL with the sensitivity of 55% and specificity of 77.5% (area under curve (AUC) = 0.690, 95% confidence interval (CI): 0.574–0.806, *P* < 0.01, Fig. 2), and the power of the test was 0.86. Besides, the MPV level was 9.55 fL with sensitivity of 75% and specificity of 70% (AUC = 0.733, 95% CI: 0.620–0.846, *P* < 0.001, Fig. 3), and the power of the test was 0.97.

### Comparison of platelet function and parameters between subgroups of KD patients

No statistically significant difference observed regarding platelet function and platelet parameters between the IVIG resistance and response groups with KD both in the febrile and defervescence periods (*P* > 0.05, Table 2). Meanwhile, a similar result was seen between the CAA and the KD without CAA groups (Table 3).

**Table 2:** The clinical and laboratory data between the IVIG resistance and response groups with KD.

Patient characteristics	Febrile period			Defervescence period		
	IVIG Resistance (n = 3)	IVIG Response (n =37)	P	IVIG Resistance (n = 3)	IVIG Response (n =37)	P
WBC (×10^9^ cells/L)	12.27 ± 3.4	13.33 ± 3.12	0.576	9.60 ± 3.08	7.22 ± 2.62	0.143
HB (g/L)	115.67 ± 2.89	111.08 ± 9.03	0.391	106.67 ± 9.50	105.73 ± 10.06	0.877
PLT (×10^9^ cells/L)	306.67 ± 10.26	335.11 ± 107.63	0.654	363.00 ± 59.76	442.65 ± 136.83	0.328
PDW (fL)	9.57 ± 1.08	10.21 ± 1.45	0.463	9.5 ± 1.00	10.04 ± 1.31	0.494
MPV (fL)	9.27 ± 0.21	9.34 ± 0.78	0.872	9.00 ± 0.26	9.37 ± 0.76	0.412
PCT (%)	0.28 ± 0.01	0.33 ± 0.13	0.546	0.32 ± 0.04	0.41 ± 0.12	0.218
CRP (mg/dL)	110.2 ± 69.49	80.15 ± 39.49	0.239	56.00 ± 41.90	34.46 ± 28.30	0.228
ESR (mm/hour)	66.07 ± 14.01	66.18 ± 25.26	0.974	—	—	—
EPI (s)	97 ± 10.58	100.68 ± 21.48	0.773	146.67 ± 34.53	167.45 ± 46.50	0.341
ADP (s)	80.67 ± 8.74	80.22 ± 16.70	0.964	88.67 ± 2.89	98.00 ± 20.16	0.433

KD = Kawasaki disease, IVIG = intravenous immunoglobulin, WBC = white blood cell, HB = hemoglobin, PLT = platelet count, PDW = platelet distribution width, MPV = mean platelet volume, PCT = platelet hematocrit, CRP = C-reactive protein, ESR = erythrocyte sedimentation rate, EPI = closure time of collagen and epinephrine, ADP = closure time of collagen and adenosine diphosphate.

**Table 3:** The clinical and laboratory data in KD patients with and without CAAs.

Patient characteristics	Febrile period			Defervescence period		
	KD with CAA (n = 4)	KD without CAA (n =36)	P	KD with CAA (n = 4)	KD without CAA (n = 36)	P
WBC (×10^9^ cells/L)	13.15 ± 4.47	13.26 ± 3.00	0.948	10.55 ± 3.41	7.44 ± 3.19	0.074
HB (g/L)	109.75 ± 12.74	111.61 ± 8.47	0.693	98.25 ± 22.69	106.36 ± 8.04	0.528
PLT (×10^9^ cells/L)	396.25 ± 239.17	325.94 ± 81.08	0.202	488.50 ± 225.75	442.61 ± 122.59	0.519
PDW (fL)	10.28 ± 1.69	10.14 ± 1.42	0.865	9.68 ± 1.28	9.95 ± 1.24	0.673
MPV (fL)	9.28 ± 1.03	9.34 ± 0.73	0.868	9.08 ± 0.71	9.33 ± 0.71	0.501
PCT (%)	0.35 ± 0.17	0.32 ± 0.12	0.696	0.43 ± 0.16	0.41 ± 0.11	0.727
CRP (mg/dL)	89.75 ± 50.59	81.67 ± 41.65	0.721	54.75 ± 51.87	33.92 ± 25.24	0.483
ESR (mm/hour)	74.5 ± 19.28	65.19 ± 25.01	0.479	—	—	—
EPI (s)	105.25 ± 13.33	99.86 ± 21.54	0.629	162.25 ± 26.99	158.22 ± 45.65	0.865
ADP (s)	78.25 ± 14.36	80.47 ± 16.53	0.798	95.25 ± 10.11	95.06 ± 21.46	0.986

KD = Kawasaki disease, CAA = coronary artery abnormality, WBC = white blood cell, HB = hemoglobin, PLT = platelet count, PDW = platelet distribution width, MPV = mean platelet volume, PCT = platelet hematocrit, CRP = C-reactive protein, ESR = erythrocyte sedimentation rate, EPI = closure time of collagen and epinephrine, ADP = closure time of collagen and adenosine diphosphate.

No significant differences showed in PDW and MPV between the febrile period and convalescence stage of KD (*P* > 0.05, Table 4). However, PLT and PCT were statistically higher in the defervescence period than those in the other stages of KD (*P* < 0.05, Table 4). Additionally, both EPI and ADP were statistically higher in the febrile period than those in the defervescence period (*P* < 0.001, Table 4).

**Table 4:** The clinical and laboratory data of KD patients in the acute febrile phase (0–1 week), defervescence period (1–2 weeks), and convalescence stage (4–8 weeks).

Patients characteristics	0–1 week (n = 40)	1–2 weeks (n = 40)	4–8 weeks (n = 37)	P
WBC (×10^9^ cells/L)	13.25 ± 3.11	7.75 ± 3.31	6.83 ± 2.00	<0.001
HB (g/L)	111.43 ± 8.78	105.55 ± 10.19	118.56 ± 10.57	<0.001
PLT (×10^9^ cells/L)	332.98 ± 103.71	447.20 ± 132.67	356.44 ± 104.57	<0.001
PDW (fL)	10.16 ± 1.43	9.93 ± 1.23	9.99 ± 1.41	0.732
MPV (fL)	9.34 ± 0.75	9.31 ± 0.71	9.36 ± 0.62	0.957
PCT (%)	0.32 ± 0.13	0.41 ± 0.12	0.33 ± 0.09	0.002
CRP (mg/dL)	82.52 ± 41.97	36.92 ± 28.37	3.95 ± 2.42	<0.001
EPI (s)	100.4 ± 20.80	158.67 ± 43.7	—	<0.001
ADP (s)	80.25 ± 16.17	95.08 ± 20.53	—	<0.001

WBC = white blood cell, HB = hemoglobin, PLT = platelet, PDW = platelet distribution width, MPV = mean platelet volume, PCT = platelet hematocrit, CRP = C-reactive protein, EPI = closure time of collagen and epinephrine, ADP = closure time of collagen and adenosine diphosphate.

## Discussion

In this study, we prospectively investigate the platelet function with the PFA-200 and platelet parameters during the various stages of KD. In previous studies, PFA has been widely used for monitoring of antiplatelet drugs^18^, von Willebrand disease screening^19^, and the management of surgical bleeding risk^20^. To the best of our knowledge, this study is the first to assess the platelet function using the PFA-200 in children with KD.

The results of our research indicated that EPI in the KD group showed no significant difference during the febrile period than that in the febrile control group but was significantly higher than that in the afebrile group. On the other hand, ADP showed a statistically significant difference between the two periods in the KD group and the two control groups. Besides, all the febrile patients including those in the KD and control groups were administered oral ibuprofen at a dose of 5–10 mg/kg when their temperature rose above 38.5 °C, with 4–7 doses/a week. According to the literature, ibuprofen can prolong EPI in around 95% of healthy people but has little or no effect on ADP^21^. Besides, abnormally prolonged EPI returns to normal within 24 hours following cessation of ibuprofen^22^. Therefore, differences in EPI between the three groups in the febrile period may be attributed to the effect of ibuprofen, and the use of aspirin is considered a reason for the higher EPI in the defervescence period of KD.

During aspirin therapy, the EPI value was affected, whereas the ADP should not have been affected^23^. It is worth noting that endothelial damage was detected in KD, which could lead to the release of von Willebrand factor (vWF); platelets activated by vWF binding further enhance coagulation by acting as a scaffold for intrinsic coagulation^24^. Theoretically, the ADP value should reduce in a high coagulation state. However, our study unexpectedly observed that ADP in the defervescence period of KD was slightly prolonged compared with that in the febrile period of KD or that in the defervescence period of the febrile control group. Except for other factors, such as the reduced hematocrit or platelet count^25^, it indicates that IVIG can inhibit platelet activation in KD patients, as reported in previous studies^26,27^.

Over the years, LTA is considered as the gold standard for diagnosing platelet dysfunction^28^, which can detect defects in platelet secretion and adhesion^29^. However, it is not only time-consuming but also requires a specialized laboratory, which makes it difficult to widespread clinical application. Compared with LTA, the PFA-200 program is much simpler and faster, using disposable cartridges for testing, less affected by analytical procedures, and does not require a specialized laboratory. Besides, the PFA-200 detects platelet adhesion under the influence of shear force and is more sensitive than the detection of platelet secretion dysfunction^29^. Moreover, it should be noted that only one study^30^ assessed platelet adhesion function in KD by Baumgartner’s method published nearly 30 years ago, which was investigated platelet function using an annular perfusion chamber^31^, found decreased platelet adhesion in KD patients treated with IVIG. Our results were similar to those in this paper, which was suggested that PFA-200, as a new reliable and straightforward method, could be used to detect platelet function, especially platelet adhesion function, of KD patients in the future. More experiments are needed to confirm this conclusion.

For a long time, there were several acute inflammatory biomarkers, such as ESR, WBC, and CRP, used in conjunction with the clinical characteristics for the diagnosis of KD^32^. However, a gold standard biomarker for the identification of KD is still lacking. Platelets are commonly thought to play a role in hemostasis and thrombosis^33^, but their role in immune responses and inflammation has attracted increasing attention^34^. PDW is a measure of platelet size variability and increases with platelet activation^35^. Furthermore, PDW is considered to be a more specific platelet reactivity indicator than MPV because it is not affected by the individual platelet distention caused by platelet swelling^36^. MPV is an indicator of platelet activity and size^37^, which can be easily and economically measured by automated hematology analyzers^38^. Due to increased platelet size and volume can reflect thrombotic and inflammatory conditions^37^, MPV is considered as a possible marker of platelet function and activation^39^.

Previous studies have shown that PDW and MPV can be used as markers for cardiovascular risk^40^. Liu *et al*.^41^ reported that KD patients have lower PDW and MPV, but those were not useful markers for predicting CAA. Similarly, we found lower PDW and MPV in KD children, which could be helpful in the diagnosis of KD. However, still, PDW and MPV were not useful to identify KD patients with CAAs or IVIG resistance in the present study. The decreased platelet volume may be due to the consumption or isolation of large activated platelets in the vascular system^42^. Igarashi *et al*.^43^ indicated that some markers, such as interleukin-6, granulocyte colony-stimulating factor, and macrophage colony-stimulating factor, increase during the acute phase of KD, which might contribute to decreasing the platelet volume of KD patients. Overall, the mechanism for the decrease in PDW and MPV in KD patients remains unclear^44^. Further research is needed to determine the exact mechanisms of low levels of PDW and MPV in KD children.

Although some reports recognized both thrombocytopenia and significant thrombocytosis as CAA or IVIG resistance predictors, the majority of studies showed no association^45,46^. The exact mechanism of thrombocytosis is unclear. It has been suggested that the elevated thrombopoietin level caused by acute inflammatory responses can lead to thrombocytopoiesis^47^. PCT is the volume percentage of platelets in the whole blood^48^, which is positively correlated with PLT and MPV. A meta-analysis indicated that PCT was associated with CAA^49^. In our study, we found higher PLT in the defervescence period of KD patients than that in the control group. However, no significant difference in the level of PLT and PCT between IVIG resistance or CAA with KD and other KD patients. The most probable reason to explain this phenomenon was that the sample size was relatively small^50^, which requires further study of more patients and longer follow-up time to clarify the relationship between IVIG resistance or CAA with KD and other KD patients.

In conclusion, our study is the first to provide a longitudinal study of platelet function changes in KD patients using PFA-200, which mimics the characteristics of platelet function *in vivo*. Inconsistent with the previous studies^24,51,52^, the platelet function did not change significantly in the febrile period of children with KD, but it weakened in the defervescence phase of KD. Besides, lower PDW and MPV in the febrile period may be available markers for the early diagnosis of KD. Further clinical trials with larger sample size are needed to confirm the significance of platelet function and parameters in KD patients.

## Materials and Methods

### Study design

We prospectively enrolled and obtained blood samples from 120 pediatric patients who were hospitalized at the Department of Pediatrics in West China Second University Hospital of Sichuan University from May 2018 to October 2018, including 40 children with KD, 40 cases as the febrile control group, and 40 as the afebrile control group. In the KD group, all patients met the definition of KD based on the American Heart Association (AHA) criteria^1^. The patients in the febrile control group were children with common fever; the diseases were mainly infectious mononucleosis, febrile pneumonia, or acute tonsillitis. The children in the afebrile control group had either afebrile pneumonia, acute gastroenteritis, or acute bronchitis. In all groups, patients were excluded if meeting any of the following criteria: (1) history of special diseases, including primary coagulation disorder or severe infection, anemia, and/or thrombocytopenia; (2) history of special medication: anticoagulation, antiplatelet drugs, blood products such as blood plasma or platelet infusion, and glucocorticoid hormone.

All KD patients were treated with oral aspirin (30 mg/kg/day) and IVIG (2 g/kg/day) in the acute phase as the initial treatment. Subsequently, low-dose aspirin (3–5 mg/kg/day) was started after the child had been afebrile for 48 to 72 hours, and continued until the patient had no evidence of coronary changes at 6 to 8 weeks after the onset of the illness, or was continued indefinitely for children who developed coronary abnormalities^1^. IVIG resistance was defined as persistent fever lasting over 36 hours after the completion of IVIG or recrudescent fever associated with KD symptoms after a defervescence period; retreatment with IVIG 2 g/kg was then performed^3^. Patients in the two control groups were conventionally treated according to their disease types.

Echocardiography was performed in the acute febrile phase (within one week after onset of illness), the defervescence period (1–2 weeks after onset of illness), and the convalescence stage (4–8 weeks after onset of illness). The diagnosis of CAA was based on the Japanese Kawasaki Disease Research Committee^53^.

Blood samples were obtained during the acute febrile phase before treatment with the first IVIG and during the defervescence period about 2–3 days after IVIG in all KD patients. An additional blood sample was obtained during the defervescence period after retreatment with IVIG in IVIG resistance KD children. In the febrile control group, blood samples were obtained during the acute febrile period and the defervescence period in patients. Additionally, blood samples were obtained only one time during the hospitalization in the afebrile control group. The baseline characteristics and laboratory data were collected at the same time, including white blood cell (WBC), hemoglobin (HB), platelet count (PLT), platelet distribution width (PDW), mean platelet volume (MPV), platelet hematocrit (PCT), C-reactive protein (CRP), and erythrocyte sedimentation rate (ESR).

We followed all KD patients for 8 weeks from the onset of the illness to obtain echocardiography and laboratory data including WBC, HB, PLT, PDW, MPV, PCT, and CRP from the hospital database. The platelet function could not be performed as it was too difficult to obtain blood samples after the children had been discharged.

### Sample collection and PFA-200 assays

The whole blood sample (2.7 ml) was collected in a sodium citrate tube containing 0.105 M citrate (3.2%) (BD Vacutainer Systems, Plymouth, Devon, United Kingdom) by venipuncture using a 21 G needle gauge. All blood samples underwent measurement of the CT value of EPI and ADP from PFA-200 within 2 hours of collection^54^. PFA-200 assays were performed according to the manufacturer’s instructions. The same batch of each test cartridge was used throughout the entire study. Cartridges were allowed to warm up to room temperature before use. Next, 800 µl of blood was pipetted into the sample reservoir of each cartridge on the carousel holder before being loaded into the device. Real-time data were automatically printed out. The reference range of the EPI cartridge was based on values for 309 healthy unmedicated subjects and was 82–150 s when the blood was collected in tubes containing 0.105 M citrate (3.2%, package insert); whereas that for the ADP cartridge was 62–100 s using the same conditions^55^. Maximal CT was at 300 s, and values >300 s were considered invalid.

### Statistical analyses

The statistical analyses were performed using SPSS 22.0 (IBM Corp, Armonk, NY). All continuous variables were reported as the mean ± standard deviation. The chi-square test was used to compare the frequencies between groups. The receiver operating characteristic curve was utilized to examine the predictive value of platelet function and platelet parameters in patients with KD. The area under the curve was calculated. The cutoff value from the curve used the Youden index (sensitivity + specificity–1) to identify. Differences in continuous variables among groups were assessed using the independent sample t-test or the analysis of variance (ANOVA). The results were considered to indicate statistical significance if P values were less than 0.05. Additionally, we used the PASS software version 15 (NCSS, Kaysville, UT, USA) to calculate the sample size and the power of the test.

### Ethic statement

This study was approved by the Human Use Ethical Committee of West China Second University Hospital of Sichuan University, and written informed consents were obtained from the parents or guardians of all patients. All methods were performed in accordance with the Declaration of Helsinki and the relevant guidelines.

## Data Availability

The authors confirm that all data underlying the findings are fully available without restriction. All relevant data are contained within the paper.
